# Peripheral and central effects of γ-secretase inhibition by semagacestat in Alzheimer’s disease

**DOI:** 10.1186/s13195-015-0121-6

**Published:** 2015-06-10

**Authors:** Rachelle S Doody, Rema Raman, Reisa A Sperling, Eric Seimers, Gopalan Sethuraman, Richard Mohs, Martin Farlow, Takeshi Iwatsubo, Bruno Vellas, Xiaoying Sun, Karin Ernstrom, Ronald G Thomas, Paul S Aisen, for the Alzheimer’s Disease Cooperative Study

**Affiliations:** Alzheimer’s Disease and Memory Disorders center, Department of Neurology, Baylor College of Medicine, 1977 Butler Blvd, Suite E5.101 Houston, TX USA; Division of Biostatistics and Bioinformatics, Department of Family and Preventive Medicine, Department of Neurosciences, University of California San Diego, 9500 Gilman Drive, M/C 0949, La Jolla, CA 92093 USA; Department of Neurosciences, University of California San Diego, 9500 Gilman Drive, M/C 0949, La Jolla, CA, 92093 USA; Department of Neurology, Harvard Medical School, 220 Longwood Avenue, Goldenson Building, Room 420, Boston, MA 02115 USA; Eli Lilly & Company, Lilly Corporate Center, Indianapolis, IN 46285 USA; Department of Neurology, Indiana University, Indiana Alzheimer Disease Center, 355 W. 16th Street, Suite 4700, Indianapolis, IN 46202 USA; Graduate School of Medicine, University of Tokyo, 7-3-1, Hongo, Bunkyo-ku, Tokyo, 113-8654 Japan; Gerontopole UMR INSERM 1027, CHU, University of Toulouse, Toulouse, France; Biostatistics Research Center, Department of Family and Preventive Medicine, University of California San Diego, 9500 Gilman Drive, M/C 0949, La Jolla, CA 92093 USA; Alzheimer’s Disease Cooperative Study, Department of Family and Preventive Medicine, Department of Neurosciences, University of California San Diego, 9500 Gilman Drive M/C 0949, La Jolla, CA 92093 USA

## Abstract

**Introduction:**

The negative efficacy study examining the γ-secretase inhibitor semagacestat in mild to moderate Alzheimer’s disease (AD) included a number of biomarkers of the disease as well as safety outcomes. We analyzed these data to explore relationships between drug exposure and pharmacodynamic effects and to examine the correlations among outcome measures.

**Methods:**

The study was a multicenter, randomized, placebo-controlled trial of two dose regimens of semagacestat and a placebo administered for 18 months to individuals with mild to moderate AD. Changes in measures of central and peripheral drug activity were compared between the three treatment groups using one-way analysis of variance. The relationship between changes in each of the outcome measures and measures of drug exposure and peripheral pharmacodynamic effect were assessed using Spearman’s correlation coefficient.

**Results:**

Assignment to the active treatment arms was associated with reduction in plasma amyloid-β (Aβ) peptides, increase in ventricular volume, decrease in cerebrospinal fluid phosphorylated tau (p-tau) and several other laboratory measures and adverse event categories. Within the active arms, exposure to drug, as indicated by area under the concentration curve (AUC) of blood concentration, was associated with reduction in plasma Aβ peptides and a subset of laboratory changes and adverse event rates. Ventricular volume increase, right hippocampal volume loss and gastrointestinal symptoms were related to change in plasma Aβ peptide but not AUC, supporting a link to inhibition of γ-secretase cleavage of the amyloid precursor protein. Cognitive decline correlated with ventricular expansion and reduction in p-tau.

**Conclusion:**

These findings may inform future studies of drugs targeting secretases involved in Aβ generation.

**Trial registration:**

ClinicalTrials.gov Identifier: NCT00594568. Registered 11 January 2008.

**Electronic supplementary material:**

The online version of this article (doi:10.1186/s13195-015-0121-6) contains supplementary material, which is available to authorized users.

## Introduction

One leading theory of the pathogenesis of Alzheimer’s disease (AD) considers the sequential cleavage of the amyloid precursor protein (APP) by β- and γ-secretases to release amyloidogenic peptides to be the initiating and driving event in this neurodegenerative condition [1]. Inhibition of secretases has therefore been a major strategy in efforts to develop disease-modifying treatments for AD [2]. The strategy of γ-secretase inhibition to slow disease progression is further supported by the finding that the most common mutations that cause familial autosomal dominant AD involve presenilin, a γ-secretase component [3].

The first large-scale study of a γ-secretase modulating drug, flurbiprofen, was ineffective [4]. This failure has been attributed to insufficient pharmacodynamic effects in the brain. More recently, novel γ-secretase inhibitors have demonstrated target engagement, as indicated by reduction in cerebrospinal fluid (CSF) levels of amyloid peptides [5,6]. A major concern about this drug class has been adverse effects related to impact on non-target substrates [6-8]. In particular, Notch cleavage by γ-secretase may be inhibited by such drugs [9], with adverse effects on the gastrointestinal, immune and cutaneous systems.

Semagacestat is generally referred to as a γ-secretase inhibitor, but we note that it increases levels of amyloid-β peptide 42 (Aβ42) in the blood at low concentrations, suggesting that it may exert its activity at an allosteric site rather than the active site of the enzyme. It does not increase generation of shorter peptides (such as Aβ38), which is a characteristic of drugs referred to as γ-secretase modulators.

Semagacestat was the first γ-secretase inhibitor to reach Phase III testing in AD. The development of this drug was spurred by strong evidence for a central pharmacodynamic effect in a study in which researchers used stable isotope labeling with CSF sampling to determine the kinetics of amyloid peptide production [7]. The phase III trial, the primary results of which are reported elsewhere [10], was terminated before planned completion because of evidence of cognitive and other adverse effects in the active treatment group. Data derived from the trial afford an opportunity to evaluate the peripheral laboratory and clinical effects and the central effects of γ-secretase inhibition in AD. This experience may inform other ongoing efforts to target this enzyme complex.

## Methods

### Patients, drug dosing and blinding

The present study was approved by the institutional review boards at each participating site (see Additional file 1). Subjects aged 55 years or older with mild to moderate AD (Mini Mental State Examination (MMSE) [11] score between 16 and 26 at screening) who met National Institute of Neurological and Communicative Disorders and Stroke/Alzheimer’s Disease and Related Disorders Association criteria [12] and were in good general health and free of depression (Geriatric Depression Scale score ≤6) [13]) were randomized to escalate to 100 mg once daily or 140 mg once daily of semagacestat or placebo over 76 weeks using a triple dummy to blind dosage groups. Doses were titrated as follows: 60 mg for 2 weeks, then 100 mg; or 60 mg for 2 weeks, 100 mg for 2 weeks, then 140 mg. At baseline, subjects could be untreated or treated with background cholinesterase inhibitors with or without memantine, as long as they had been on the drug for at least 4 weeks and the doses of antidementia drugs were stable for at least 2 months. All subjects signed informed consent forms prior to participating in study procedures.

### Cognitive measures

Patients were assessed with the 11-item version of the Alzheimer’s Disease Assessment Scale–Cognitive Subscale (ADAScog11) [14] at baseline and weeks 12, 28, 40, 52, 64, 76 and 88 or at early termination and with the MMSE at screening, baseline and weeks 52, 76 and 88 or early termination.

### Biological markers and imaging outcome measures

Patients were genotyped for apolipoprotein E polymorphisms. Special lymphocyte hematology was done at baseline and weeks 12, 28, 40, 52, 64, 76 and 88 or early termination. Plasma Aβ was assessed at baseline and weeks 6, 12 and 52 or early termination. Optional CSF analysis for Aβ peptides and tau and phosphorylated tau (p-tau) proteins was conducted in willing subjects. Volumetric magnetic resonance imaging (vMRI), amyloid imaging with florbetapir (AV45) fludeoxyglucose positron emission tomography (FDG-PET), and CSF analyses were performed at baseline and week 76 or early termination. Additional details regarding methods are provided in the Appendix to the article describing the primary results of the phase III trial [10].

### Pharmacokinetic measures

Pharmacokinetic (PK) samples were collected at week 6, 12 and 52. Population PK analysis was performed using a nonlinear mixed-effects model. The model estimated individual clearance values for each subject using all concentrations collected at each visit, taking into account time from dose, the dose level administered, and estimated residual error that resulted from assay error, inaccurate sample time information or inaccurate dosing information. The clearance estimate for each individual was used to calculate area under the concentration curve (AUC) using standard equations. A similar process was undertaken to generate maximum concentration (C_max_) using standard equations to calculate C_max_ and individual estimates for various PK parameters.

### Alzheimer’s Disease Cooperative Study Data Analysis and Publication Committee

The Data Analysis and Publication Committee (DAPC) was funded by a grant from Eli Lilly to the University of California at San Diego as fiduciary for the Alzheimer’s Disease Cooperative Study (ADCS) after the semagacestat phase III studies were halted, but before the datasets were transferred to the ADCS. The timing of the contract was designed to remove any concern that payment for the work of data analysis and publication would be dependent upon the outcome. The DAPC developed a document of governance that specifies voting members of the committee, as well as non-voting members, who include a limited number of Eli Lilly employees who are familiar with the study and the data and one non-voting representative each of the Data Safety Monitoring Committee and the National Institute on Aging. The DAPC also developed a data dissemination plan and kept the ADCS Steering Committee apprised of its progress by formal reports at each Steering Committee meeting. The final publication was developed by the committee and approved by the voting members of the DAPC committee and the ADCS steering committee, and non-voting DAPC members gave feedback but did not have veto power.

### Statistical analysis

Changes in outcome measures reflecting central and peripheral activity of semagacestat were compared between the three treatment groups using one-way analysis of variance. Analyses included all randomized subjects with available data. Annualized change from baseline in cognitive, CSF and three imaging outcomes (vMRI, FDG-PET and AV45) were calculated based on the last available observation in the initial treatment period. The outcome for plasma Aβ used percentage change from baseline to 6 hours postdose at week 52. Laboratory measures included change from baseline in uric acid, albumin and eosinophils at week 76. Specific adverse events, including gastrointestinal, skin disorder, skin cancer and infection incidence, were compared between the three treatment groups using the Fisher-Freeman-Halton exact test. The relationship between change in each of the outcome measures (cognitive tests, plasma Aβ, CSF assays, vMRI, FDG-PET and rates of specific adverse events) and measures of drug exposure (AUC in the active treatment group) and peripheral pharmacodynamic effects (changes in plasma Aβ) were assessed using Spearman’s correlation coefficient [15]. R version 2.14.1 statistical software [16] was used for all statistical analyses. We did not employ corrections for multiple comparisons in this exploratory analysis.

## Results

### Outcomes by treatment group

**Figure 1:**
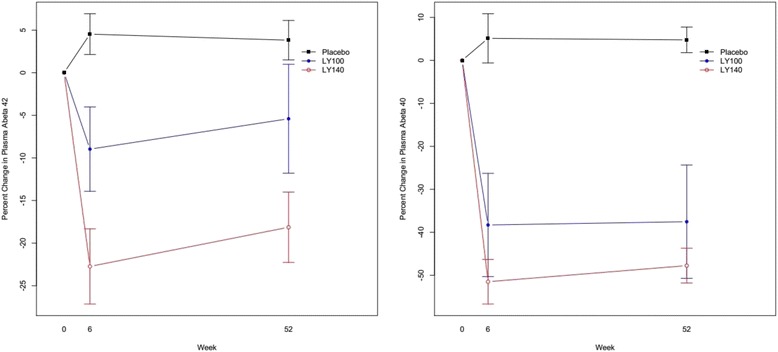
**Change in plasma amyloid-β peptide levels.** Abeta, Amyloid-β; LY100mg, Semagacestat 100 mg; LY140mg, Semagacestat 140 mg.

Summary statistics of key outcomes are shown in Table 1 for the three arms of the trial. Treatment assignment was associated with ventricular volume (greater ventricular expansion in high-dose arm), CSF p-tau (increase in placebo arm, reduction in treatment arms), plasma Aβ peptides (dose-related reduction in active arms) and several laboratory measures and adverse event categories (Table 1). The relationship between arm assignment and change in plasma Aβ peptides is shown in Figure 1. Levels of both species of amyloid peptide had declined in both active treatment arms by week 6, and the reductions were sustained until week 52 (the final analysis time point for plasma amyloid peptides).

**Table 1:** **Outcomes by treatment arms** ^**a**^

	**Placebo**	**LY 100 mg**	**LY 140 mg**	***P*** **-value**
Annualized change in ADASCog11	4.75 ± 21.92 (n = 485)	6.57 ± 25.57 (n = 482)	5.48 ± 21.92 (n = 495)	0.408
Annualized change in MMSE	−2.19 ± 3.65 (n = 396)	−2.56 ± 3.65 (n = 324)	−2.92 ± 3.65 (n = 303)	0.159
Annualized change in FDG-PET SUVR	−0.06 ± 0.07 (n = 40)	−0.13 ± 0.21 (n = 42)	−0.1 ± 0.08 (n = 33)	0.109
Annualized change in AV45 SUVR	0.05 ± 0.11 (n = 18)	0.02 ± 0.24 (n = 23)	0.07 ± 0.23 (n = 18)	0.794
Annualized change in ventricular volume	4.07 ± 3.52 (n = 80)	4.19 ± 3.92 (n = 74)	5.68 ± 4.68 (n = 67)	0.033
Annualized change in right hippocampal volume	−86.28 ± 73.86 (n = 74)	−91.08 ± 102.1 (n = 68)	−90.98 ± 71.1 (n = 64)	0.926
Annualized change in left hippocampal volume	−68.85 ± 59.01 (n = 74)	−70.4 ± 90.81 (n = 68)	−91.0 ± 101.2 (n = 64)	0.244
Annualized change in CSF Aβ40	87.66 ± 847 (n = 10)	−10.96 ± 1151 (n = 19)	−599 ± 1786 (n = 18)	0.328
Annualized change in CSF Aβ42	10.96 ± 87.66 (n = 10)	−40.18 ± 109.6 (n = 19)	−36.5 ± 182.7 (n = 18)	0.619
Annualized change in p-tau	10.96 ± 7.31 (n = 10)	−7.31 ± 14.61 (n = 19)	−3.65 ± 10.96 (n = 18)	0.009
Annualized change in total tau	94.97 ± 116.9 (n = 10)	−43.83 ± 219.2 (n = 17)	40.18 ± 127.8 (n = 18)	0.101
Percentage change in plasma Aβ40 at week 52	4.77 ± 26.67 (n = 307)	−37.54 ± 109.3 (n = 264)	−47.76 ± 32.06 (n = 243)	<0.001
Percentage change in plasma Aβ42 at week 52	3.82 ± 20.75 (n = 309)	−5.4 ± 53.1 (n = 265)	−18.14 ± 33.0 (n = 245)	<0.001
Change in uric acid at week 76	0.18 ± 0.83 (n = 210)	−0.96 ± 1.08 (n = 179)	−0.88 ± 1.22 (n = 147)	<0.001
Change in albumin at week 76	0 ± 0.25 (n = 210)	−0.09 ± 0.31 (n = 179)	−0.09 ± 0.3 (n = 147)	0.002
Change in eosinophils at week 76	−0.001 ± 0.09 (n = 207)	0.05 ± 0.17 (n = 179)	0.06 ± 0.14 (n = 143)	<0.001
Gastrointestinal symptoms	153 (31.5%)	169 (34.9%)	193 (38.8%)	0.057
Skin disorder incidence	105 (21.6%)	220 (45.4%)	269 (54%)	<0.001
Skin cancer incidence	8 (1.7%)	51 (10.5%)	56 (11.2%)	<0.001
Infection incidence	156 (32.1%)	188 (38.8%)	220 (44.2%)	<0.001
AUC		5,316 ± 1,525 (n = 480)	7,235 ± 2233 (n = 494)	<0.001
C_max_		1,105 ± 260 (n = 480)	1,508 ± 384 (n = 494)	<0.001

^a^Aβ40, Amyloid-β peptide 40; Aβ42, Amyloid-β peptide 42; ADAScog11, Alzheimer’s Disease Assessment Scale–Cognitive Subscale, 11-item version; AUC, Area under the curve; AV45, Florbetapir; C_max_, Maximum concentration; CSF, Cerebrospinal fluid; FDG-PET, Fludeoxyglucose positron emission tomography; LY, Semagacestat; MMSE, Mini Mental State Examination; p-tau, Phosphorylated tau; SUVR, Standard uptake value ratio.

As expected, the mean AUC and C_max_ increased with increasing dose, although there was overlap between the dose groups.

### Correlational analyses

Among adverse events, correlational analyses suggested that gastrointestinal symptoms, rash and skin cancer may be related to each other, while uric acid reduction, albumin reduction and infection formed a cluster (Table 2). Among markers of central nervous system effects, decline in cognition measured either by increase in ADAScog11 score or decrease in MMSE score correlated with each other and with ventricular expansion (Table 3). Consistent with the overall groupwise effects, there was an unexpected correlation between reduction in CSF p-tau and cognitive decline.

**Table 2:** **Correlations between peripheral biomarkers** ^**a**^

	**AUC**	**Plasma Aβ40**	**Uric acid**	**Albumin**	**EOS**	**GI**	**Skin**	**Skin cancer**	**Infection**
AUC	1; 0	−0.36; 0.001	−0.15; 0.007	−0.14; 0.01	0.18; 0.001	0.02; 0.65	−0.02; 0.63	−0.01; 0.81	0.14; 0.001
Plasma Aβ40		1; 0	−0.01; 0.86	0.131; 0.03	−0.19; 0.002	−0.10; 0.03	−0.08; 0.06	−0.08; 0.09	−0.07; 0.11
Uric acid			1; 0	0.08; 0.13	−0.23; 0.001	0.03; 0.60	−0.12; 0.04	−0.08; 0.13	−0.11; 0.04
Albumin				1; 0	−0.22; 0.001	0.05; 0.42	0.02; 0.72	0.02; 0.68	−0.15; 0.006
EOS					1; 0	−0.01; 0.85	0.04; 0.50	0.11; 0.06	0.08; 0.17
GI						1; 0	0.11; 0.001	0.05; 0.16	0.17; 0.001
Skin							1; 0	0.14; 0.001	0.13; 0.001
Skin cancer								1; 0	0.123; 0.001
Infection									1; 0

^a^Aβ40, Amyloid-β peptide 40; AUC, Area under the concentration curve; EOS, Eosinophils; GI, Gastrointestinal. Data are Spearman’s correlation coefficients and *P*-values (*r*; *P*).

**Table 3:** **Correlations between changes in central biomarkers** ^**a**^

	**Ventricular volume**	**CSF p-tau**	**CSF Aβ42**	**ADAScog11**	**MMSE**
FDG-PET SUVR	−0.43; 0.007	−0.14; 0.79	0.37; 0.47	−0.11; 0.36	0.23; 08
Ventricular volume	1; 0	−0.14; 0.62	−0.12; 0.67	0.46; 0.001	−0.54; 0.001
CSF p-tau		1; 0	0.37; 0.49	−0.40; 0.02	0.33; 0.05
CSF Aβ42			1; 0	−0.12; 0.48	0.31; 0.08
ADAScog11				1; 0	−0.58; 0.001

^a^Aβ42, Amyloid-β peptide 42; ADAScog11, Alzheimer’s Disease Assessment Scale–Cognitive Subscale, 11-item version; CSF, Cerebrospinal fluid; FDG-PET, Fludeoxyglucose positron emission tomography; MMSE, Mini Mental State Examination; p-tau, Phosphorylated tau; SUVR, Standard uptake value ratio. Data are Spearman’s correlation coefficients and *P*-values (*r*; *P*).

To determine whether clinical and laboratory effects were more closely associated with drug exposure or with the target pharmacodynamic effects (inhibition of γ-secretase cleavage of the APP to release amyloidogenic peptides), we examined the correlation between measures and semagacestat AUC and change in plasma Aβ40, respectively (Table 4). We used Aβ40 rather than Aβ42 because the latter values were confounded in some cases by measurement below the level of detection.

**Table 4:** **Correlations of change in selected outcome measures with drug exposure and reduction in plasma amyloid-β peptide 40 in the active arms** ^**a**^

	**Semagacestat AUC**			**Change in plasma Aβ40**			**AUC adjusted for Aβ40**			**Aβ40 adjusted for AUC**		
	***r***	**n**	***P***	***r***	**n**	***P***	***r***	**n**	***P***	***r***	**n**	***P***
Change in uric acid at week 76	−0.151	320	0.007	0.011	263	0.857	−0.149	260	0.016	0.071	260	0.255
Change in albumin at week 76	−0.14	320	0.012	−0.131	263	0.033	−0.037	260	0.548	−0.107	260	0.084
Gastrointestinal symptoms	0.015	943	0.654	0.099	507	0.025	−0.004	501	0.933	0.094	501	0.034
Skin disorder incidence	0.016	943	0.627	0.083	507	0.062	−0.048	501	0.279	0.096	501	0.031
Skin cancer incidence	−0.012	943	0.71	0.075	507	0.091	−0.039	501	0.389	0.084	501	0.059
Infection incidence	0.136	943	<0.001	0.072	507	0.101	0.068	501	0.129	0.042	501	0.344
Annualized change in FDG SUVR	0.22	74	0.06	<0.001	48	0.998	0.357	48	0.010	−0.134	48	0.364
Annualized change in ventricular volume	0.003	139	0.97	0.242	91	0.021	−0.109	91	0.302	0.264	91	0.010
Annualized change in right hippocampal volume	−0.064	130	0.472	−0.264	86	0.014	−0.039	86	0.719	−0.222	86	0.038
Annualized change in left hippocampal volume	−0.109	130	0.216	−0.036	86	0.74	−0.167	86	0.123	0.043	86	0.697
Annualized change in AV45 SUVR	0.133	40	0.414	0.316	27	0.109	−0.079	27	0.697	0.324	27	0.093
Annualized change in ADASCog11	−0.012	938	0.707	0.037	505	0.403	−0.080	499	0.074	0.060	499	0.177
Annualized change in MMSE	0.038	618	0.351	−0.019	496	0.678	0.057	490	0.211	−0.037	490	0.414
Annualized change in p-tau	0.02	37	0.909	−0.124	33	0.491	0.101	33	0.578	−0.154	33	0.395
Annualized change in total tau	0.07	35	0.691	0.003	31	0.986	−0.017	31	0.930	0.010	31	0.957
Annualized change in CSF Aβ42	−0.13	37	0.443	0.152	33	0.399	−0.103	33	0.571	0.179	33	0.318

^a^Aβ40, Amyloid-β peptide 40; Aβ42, Amyloid-β peptide 42; ADAScog11, Alzheimer’s Disease Assessment Scale Cognitive Subscale, 11-item version; AUC, Area under the concentration curve; AV45, Florbetapir; CSF, Cerebrospinal fluid; FDG-PET, Fludeoxyglucose positron emission tomography; MMSE, Mini Mental State Examination; p-tau, Phosphorylated tau; SUVR, Standard uptake value ratio.

Uric acid reduction was correlated to drug exposure but not to plasma Aβ change, suggesting a mechanism distinct from inhibition of APP cleavage such as drug-induced Fanconi syndrome. Reduction in serum albumin and increase in eosinophil counts correlated with both AUC and reduction in plasma Aβ. There was a trend relating decrease in FDG PET SUVR to AUC, but no relationship with change in plasma Aβ reducing the likelihood that the proposed pharmacological mechanism of the drug influenced FDG signal. In contrast, gastrointestinal symptoms were related to plasma Aβ change but not to drug exposure, consistent with a shared mechanism related to γ-secretase cleavage of APP. Of note, MRI volumetric change, specifically atrophy as indicated by increase in ventricular volume, was also correlated to change in plasma Aβ. Because drug exposure and change in plasma Aβ40 are strongly correlated, adjusting the correlations of each with the other generally reduces the association of each measure; however, the relationship between change in plasma Aβ40 and ventricular volume and right hippocampal volume were essentially unaffected by adjustment for AUC.

## Discussion

Semagacestat reduced plasma levels of Aβ peptides, consistent with γ-secretase inhibition in the periphery. The absence of an effect on CSF Aβ peptide levels was consistent with the findings in the phase II trial [17] and may have been related to the small numbers of lumbar punctures and their timing in relation to dosing [7,17]. Thus, unlike our previous study in which we used the stable isotope labeling kinetic technique [7], the available data from this trial do not confirm adequate target engagement in the central nervous system, clouding interpretation of the results.

The most notable central nervous system effect was an adverse effect on cognition in the high-dose arm, leading to early termination of the trial, and a reduction in change in CSF p-tau in the active arms compared with placebo [10]. The p-tau effect is difficult to interpret. Although it is consistent with a possible beneficial effect on disease pathology, suggesting a link between Aβ peptide production and downstream tau abnormalities, the fact that p-tau decreases have been found in longitudinal studies of AD [18] could also mean that this finding was associated with increased neurodegeneration due to accelerated disease. The worsening of cognition in the face of these findings suggests that a substrate of γ-secretase other than APP may have been responsible for the cognitive effects. However, p-tau increased in the placebo group more than it decreased in the treated group, which may point to some instability in the assay. In contrast, the correlational studies link the drug-related change in plasma Aβ to increased brain atrophy. Whereas such atrophy has been associated with antiamyloid immunotherapy and could be related to reduction in inflammation or other effects of amyloid removal, the present data link atrophy to an adverse effect on cognition consistent with a deleterious pharmacological effect on the disease process. Further, it must be emphasized that these analyses do not establish causality and are confounded by dependence among the various measures.

Similar adverse effects have been reported with another γ-secretase inhibitor, avagacestat [6]. Specifically, gastrointestinal and skin rash findings, non-melanoma skin cancer and worsening cognition at higher doses were observed with that drug, strongly supporting a link to γ-secretase inhibition. The development of semagacestat [19,20] has been discontinued. A number of research groups continue to pursue γ-secretase modulation and β-site APP-cleaving enzyme 1 (or BACE) inhibition as alternative, perhaps safer, routes to reduction of amyloid peptide production.

If the adverse effects are related to off-target substrates, γ-secretase modulators, which do not act at the active site of the enzyme complex and do not interfere with cleavage of non-APP substrates, may not carry these risks. But the correlational analyses seem more consistent with a direct relationship among adverse clinical effects, increase in atrophy, cognitive decline and possibly p-tau reduction.

There were correlations among peripheral and central measures of drug level and activity and adverse effects on cognition, consistent with related mechanisms. However, causality cannot be inferred; each of these effects is related to drug exposure, so correlations among them are expected. Observed drug effects could be related to inhibition of cleavage of substrates other than APP. Semagacestat is a non-specific inhibitor of γ-secretase, and the half-maximal effective concentration (EC_50_) for inhibition of Notch cleavage has been reported to be similar to that for APP cleavage [21]; other methods have indicated a tenfold stronger inhibition of Notch cleavage [22]. Presumably, the EC_50_ of semagacestat for other transmembrane proteins that are substrates for γ-secretase may be in the same range. On the basis of the clinical trial results alone, the question could be raised whether a lower dose of semagacestat given twice daily might have been better tolerated; however, toxicology studies using beagle dogs and rats showed that the same total daily dose of semagacestat was tolerated very poorly when divided into twice daily dosing due to findings consistent with Notch-mediated gastroenteropathy (data on file, Eli Lilly & Company). It also remains possible that inhibition of γ-secretase cleavage of APP is related to the adverse effect on cognition observed in the high-dose arm of this trial. Further studies of γ-secretase inhibitors and modulators should include monitoring for adverse systemic and cognitive effects.

Progression of AD is characterized by decline in cognitive performance and atrophy of brain tissue. In the semagacestat trial, high-dose treatment had an adverse effect on cognition [10], and in the present analyses, cognitive decline was related to expansion of ventricular volume. The relationship between reduction in CSF p-tau and worsening cognition among those treated with semagacestat cannot be readily explained with the available data. Treatment effects on cognitive and biomarker measures in AD may not be predicted by the patterns of longitudinal change noted in observational studies. Elucidation of the mechanisms of these discordant effects will require data derived from additional trials of various therapeutic interventions, as well as more longitudinal data on biomarker changes in mild to moderate AD.

## Conclusions

Analysis of the relationships among PK/pharmacodynamic measures, biomarkers and laboratory tests in the phase III trial of semagacestat in mild to moderate AD provides some insight into the neurobiological and clinical impact of γ-secretase inhibition. Exposure to drug was associated with reduction in plasma Aβ peptides, as well as a subset of laboratory changes and adverse event rates. Measures of brain atrophy and gastrointestinal symptoms were related to changes in plasma Aβ peptide but not drug concentration, supporting a link to inhibition of γ-secretase cleavage of the APP. Cognitive decline correlated with ventricular expansion and reduction in p-tau. These findings may be useful to future investigators in the design of studies targeting secretases involved in Aβ generation.

## Additional file

Additional file 1:
**Ethical review board information and informed consent document.**

## Abbreviations

ADAScog11: Alzheimer’s Disease Assessment Scale–Cognitive Subscale, 11-item version
Aβ: Amyloid-β
AUC: Area under the concentration curve
AD: Alzheimer’s disease
ADCS: Alzheimer’s Disease Cooperative Study
APP: Amyloid precursor protein
AV45: Florbetapir
C_max_: Maximum concentration
CSF: Cerebrospinal fluid
DAPC: Data Analysis and Publication Committee
EC_50_: Half-maximal effective concentration
EOS: Eosinophils
FDG-PET: Fludeoxyglucose positron emission tomography
GI: Gastrointestinal
LY: Semagacestat
MMSE: Mini Mental State Examination
PK: Pharmacokinetic
p-tau: Phosphorylated tau
SUVR: Standard uptake value ratio
vMRI: Volumetric magnetic resonance imaging
