# Supplementary material for: Peripheral and central effects of γ-secretase inhibition by semagacestat in Alzheimer’s disease
Source: Alzheimers Res Ther. 2015 Jun 10;7(1):36. doi: 10.1186/s13195-015-0121-6 (PMC4461930; doi:10.1186/s13195-015-0121-6)
Supplement: Additional file 1: — Ethical review board information and informed consent document. [file 13195_2015_121_MOESM1_ESM.pdf]

Ethical Review Board Information and Informed Consent Document

Approval Date: 19-Apr-2012 GMT

Investigator Name	IRB and/or IEC Name and Address
Pedro Abizanda	CEIC Hospital La Princesa, Diego de Leon 62, Madrid, Spain
Judith Aharon-Peretz	Rambam Medical Center EC, Memory Disorder Unit- ‘Beit Ha’Even’ Clinic, Ha'aliya St., Bat Galim, Haifa, Israel
Suvarna Alladi	Institutional Ethics Committee, Nizam's Institute of Medical Sciences, Panjagutta, Hyderabad, India
David Ames	DLA Phillips Fox, 140 William Street, PO BOX 4301PP, Melbourne, Australia
Niels Andreasen	Regionala Etikprövningsnämnden i Stockholm, FE 289, Stockholm, Sweden
Steven Arnold	University of Pennsylvania, Local IRB/IEC
Elissa Ash	Sourasky Medical Center EC, The Tel Aviv Sourasky Medical Center,6 Weizmann St, Tel Aviv, Israel
Sanjay Asthana	University of Wisconsin-Madison, Local IRB/IEC, Madison, WI, USA
Maria Barcikowska	Komisja Bioetyczna przy ORL Wielkopolskiej Izby Lekarskiej, Nowowiejskiego 51, Poznan, Poland
Frederick Barkhuizen	Penticton, BC, Canada
Luis Barra	Comité Ético Científico SSMO, Av. Salvador 364, Providencia, Santiago, Chile
Serge Belliard	CPP SOOM 1, Place du Dr. Baylac, Bâtiment MNH – TSA 40031, Toulouse, Haute Garonne, France
Felix Bermejo	CEIC Hospital La Princesa, Diego de Leon 62, Madrid, Spain
Irene Bettinger	St Luke Hospital IRB, 4401 Wornall Road, Kansas City, MO. USA
Leszek Bidzan	Komisja Bioetyczna przy ORL Wielkopolskiej Izby Lekarskiej, Nowowiejskiego 51, Poznan, Poland
Rafael Blesa	CEIC Hospital La Princesa, Diego de Leon 62, Madrid, Spain
Mercedes Boada	CEIC Hospital La Princesa, Diego de Leon 62, Madrid, Spain
Oscar Anibal Boga	Comité Independiente de ética para Ensayos en Farmacología Clínica, J.E. Uriburu 774-Piso 1, Ciudad Autónoma de Buenos Aire, CBA, Argentina

Investigator Name	IRB and/or IEC Name and Address
Ubaldo Bonuccelli	Azienda USL n. 12 Viareggio, via Aurelia 335, Lido di Camaiore, Italy
Michael Bowman	Copernicus IRB, USA
Guy Brannon	Copernicus IRB, USA
John Brockington	Western IRB, USA
Henry Brodaty	NSCCH Human Research Ethics Committee (Harbour & Hawkesbury), Level 4, Vindin House Royal North Shore Hospital, St. Leonards, NSW
Roger Bullock	Cambridger 4 Research Ethics Committee, Victoria House, Capital Park, FULBOURN, Cambridge, UK
James Burke	Duke University IRB, Campus Box 2712, Durham, NC, USA
Vincent Camus	CPP SOOM 1, Place du Dr. Baylac, Bâtiment MNH – TSA 40031, Toulouse, Haute Garonne, France
Fernando Castellanos	CEIC Hospital La Princesa, Diego de Leon 62, Madrid, Spain
Roger Clarnette	Hollywood Private Hospital Research Ethics Committee, Monash Avenue, Nedlands, Western Australia
Christo Coetzee	Pharma Ethics, 123 Amcor Road, Lyttleton Manor, Gauteng, South Africa
Giancarlo Comi	CE Fondazione Centro S. Raffaele del Monte Tabor (IRCCS), via Olgettina, 60, Milano, Italy
Greg Cooper	Copernicus IRB, USA
Peter Crome	Cambridger 4 Research Ethics Committee, Victoria House, Capital Park, FULBOURN, Cambridge, UK
Alfonso J. Cruz-Jentoft	CEIC Hospital La Princesa, Diego de Leon 62, Madrid, Spain
Peter Paul De Deyn	Commissie Medische Ethiek van de UZ/KULeuven, Herestraat 49, Leuven, Belgium
Charles DeCarli	University of California-Davis IRB, Davis, CA., USA
Jose DeLaGandara	Copernicus IRB, USA
Olivier Deryck	Commissie Medische Ethiek van de UZ/KULeuven, Herestraat 49, Leuven, Belgium

Investigator Name	IRB and/or IEC Name and Address
Raul Omar Dominguez	Comité Independiente de ética para Ensayos en Farmacología Clínica, J.E. Uriburu 774-Piso 1, Ciudad Autónoma de Buenos Aire, Argentina
Ranjan Duara	Copernicus IRB, USA
Tzvi Dwolatzky	Soroka Medical Center EC, 2 Hazadik from Jerusalem St., Beer Sheva, Israel
Keith Edwards	Copernicus IRB, USA
Anne Ekdahl	Regionala Etikprövningsnämnden i Stockholm, FE 289, Stockholm, Sweden
Peter Falkai	Uni Göttingen, Robert-Koch-Strasse 40, Goettingen, Germany
Arturo Famulari	Comité Independiente de ética para Ensayos en Farmacología Clínica, J.E. Uriburu 774-Piso 1, Ciudad Autónoma de Buenos Aire, Argentina
Martin Farlow	Indiana University IRB, Union Bldg 618, 620 Union Drive, Indianapolis, IN., USA
Mildred Farmer	Copernicus IRB, USA
Manuel Fernandez Martinez	CEIC Hospital La Princesa, Diego de Leon 62, Madrid, Spain
Steven Ferris	BRANY, New York, NY. USA
Patricio Fuentes	Comité Ético Científico SSMO, Av. Salvador 364, Providencia, Santiago, Chile
Maria Laura Garau	Comité Independiente de ética para Ensayos en Farmacología Clínica, J.E. Uriburu 774-Piso 1, Ciudad Autónoma de Buenos Aire, Argentina
Gary Gerard	Copernicus IRB, USA
Marcel Germain	IRB Services, 372 Hollandview Trail, Suite 300, Aurora, Ontario, Canada
Amitabha Ghosh	Institutional Ethics Committee, Apollo Gleneagles Hospitals, 58, Canal Circular Road, Kolkata, West Bengal, India
Rolando Giannaula	Comité Independiente de ética para Ensayos en Farmacología Clínica, J.E. Uriburu 774-Piso 1, Ciudad Autónoma de Buenos Aire, Argentina

Investigator Name	IRB and/or IEC Name and Address
Pedro Gil Gregorio	CEIC Hospital La Princesa, Diego de Leon 62, Madrid, Spain
Yoshinori Go	Yoshimura Naika Institutional Review Board, 3-7-16 Hirao, Chuo-ku, Fukuoka-Ken, Japan
Timo Grimmer	Uni Göttingen, Robert-Koch-Strasse 40, Goettingen, Germany
Katarzyna Gustaw	Komisja Bioetyczna przy ORL Wielkopolskiej Izby Lekarskiej, Nowowiejskiego 51, Poznan, Poland
Klaus Hager	Uni Göttingen, Robert-Koch-Strasse 40, Goettingen, Germany
Didier Hannequin	CPP SOOM 1, Place du Dr. Baylac, Bâtiment MNH – TSA 40031, Toulouse, Haute Garonne, France
Jonathan Harris	Copernicus IRB, USA
Kazunori Hashimoto	Tokushima University Hospital IRB, 2-50-1 Kuramoto-cho, Tokushima, Tokushima-Ken, Japan
Peter Høgh	Den Videnskabsetiske Komité for Region Hovedstaden, Regionsgården, Kongens Vænge 2, Hillerød, Denmark
Willis Holloway, Jr.	Oklahoma City, OK. USA
Vjera Holthoff	Uni Göttingen, Robert-Koch-Strasse 40, Goettingen, Germany
Lawrence Honig	Western IRB, USA
Jan Ilkowski	Komisja Bioetyczna przy ORL Wielkopolskiej Izby Lekarskiej, Nowowiejskiego 51, Poznan, Poland
Fraser Inglis	Cambridger 4 Research Ethics Committee, Victoria House, Capital Park, FULBOURN, Cambridge, UK
Travis Jackson	Copernicus IRB, USA
Soren Jakobsen	Den Videnskabsetiske Komité for Region Hovedstaden, Regionsgården, Kongens Vænge 2, Hillerød, Denmark
Venu Gopal Jhanwar	DMHC Ethics Committee, B27/70 MN, Durgakund, Varanasi, India
Peter Johannsen	Den Videnskabsetiske Komité for Region Hovedstaden, Regionsgården, Kongens Vænge 2, Hillerød, Denmark
Roy Jones	Cambridger 4 Research Ethics Committee, Victoria House, Capital Park, FULBOURN, Cambridge, UK

Investigator Name	IRB and/or IEC Name and Address
Ryuji Kaji	Tokushima University Hospital IRB, 2-50-1 Kuramoto-cho, Tokushima, Tokushima-Ken, Japan
Marvin Kalafer	Copernicus IRB, USA
Sebastiana Kalula	University of Cape Town - Research Ethics Committee, Groote Schuur Hospital, Cape Town, Western Cape, South Africa
Diana Kerwin	Chicago, IL. USA
Simon Kesler	Pharma Ethics, 123 Amcor Road, Lyttleton Manor, Gauteng, South Africa
Yuri Kitamura	Hospital Combination IRB, 1-14 Minamikubo, Sanyou Kochi-Building, Kochi-Shi, Kochi-Ken, Japan
Iwona Kloszewska	Komisja Bioetyczna przy ORL Wielkopolskiej Izby Lekarskiej, Nowowiejskiego 51, Poznan, Poland
Hans Kluenemann	Uni Göttingen, Robert-Koch-Strasse 40, Goettingen, Germany
Jan Kochanowicz	Komisja Bioetyczna przy ORL Wielkopolskiej Izby Lekarskiej, Nowowiejskiego 51, Poznan, Poland
Akira Kondo	Tokushima University Hospital IRB, 2-50-1 Kuramoto-cho, Tokushima, Tokushima-Ken, Japan
Janus Kremer	Comité Independiente de ética para Ensayos en Farmacología Clínica, J.E. Uriburu 774-Piso 1, Ciudad Autónoma de Buenos Aire, Argentina
David Kudrow	Copernicus IRB, USA
Susan Kurrle	NSCCH Human Research Ethics Committee (Harbour & Hawkesbury), Level 4, Vindin House Royal North Shore Hospital, St. Leonards, NSW
Andrew Larner	Cambridger 4 Research Ethics Committee, Victoria House, Capital Park, FULBOURN, Cambridge, UK
Marcolo Leiva	Comité Ético Científico SS Valdivia, Av. Simpson 850, Valdivia, Chile
Tersia Lichtenstein	IRB Services, 372 Hollandview Trail, Suite 300, Aurora, Ontario, Canada
Stan Lipschitz	Pharma Ethics, 123 Amcor Road, Lyttleton Manor, Gauteng, South Africa

Investigator Name	IRB and/or IEC Name and Address
Jarmo Liukkonen	Northern Savo Hospital District, Research Ethics Committee, PL 1777, Rakennus 4, Kuopio, Finland
Oscar Lopez	Copernicus IRB, USA
Adolfo Lopez de Munain	San Sebastian, Spain
Juan J. Lopez Lozano	CEIC Hospital La Princesa, Diego de Leon 62, Madrid, Spain
Mary Lyles	Wake Forest University IRB, USA
William Maier	Copernicus IRB, USA
Alastair Mander	Barwon Health Research and Ethics Advisory Committee, The Geelong Hospital PO Box 281, Geelong, Victoria, Australia
Facundo Manes	Comité Independiente de ética para Ensayos en Farmacología Clínica, J.E. Uriburu 774-Piso 1, Ciudad Autónoma de Buenos Aire, Argentina
David Margolin	Copernicus IRB, USA
Claudio Mariani	EC per la Sperimentazine clinica Ospedale L. Sacco, Via Giovan Battista Grassi, 74, Milano, Italy
Walter Martinez	Copernicus IRB, USA
P.S. Mathuranath	Institutional Ethics Committee, SCTIMST, Trivandrum, India
Kanji Matsumoto	Fukui General Hospital IRB, 1-42-1 Nittaduka, Fukui, Fukui-Ken, Japan
Zeev Meiner	Hadassah Har Hatzofim Medical Center EC, Kiryat Hadassah, Jerusalem, Israel
Ron Milo	Barzilai Medical Center EC, 3 Hahistadrut St., Ashkelon, Israel
Jacobo Mintzer	MUSC IRB, 19 Hagood Street, Room 601, MSC 857, Charleston, SC, USA
Lorenzo Morlan Gracia	Getafe, Madrid, Spain
Yoshihiko Nishida	Tokushima University Hospital IRB, 2-50-1 Kuramoto-cho, Tokushima, Tokushima-Ken, Japan
Margarita Nunez	Copernicus IRB, USA

Investigator Name	IRB and/or IEC Name and Address
Hasse Olofsson	Regionala Etikprövningsnämnden i Stockholm, FE 289, Stockholm, Sweden
Henrik Östlund	Regionala Etikprövningsnämnden i Stockholm, FE 289, Stockholm, Sweden
Pierre-Jean Ousset	Toulousse, France
P. Passmore	Cambridger 4 Research Ethics Committee, Victoria House, Capital Park, FULBOURN, Cambridge, UK
Jordi Peña	CEIC Hospital La Princesa, Diego de Leon 62, Madrid, Spain
Charles Pinto	The Bandra Holy Family Medical Research Society, Bandra, Mumbai, India
Steven Potkin	University of California-Irvine IRB, 5171 California Avenue, Irvine, CA. USA
Felix Potocnik	Pharma Ethics, 123 Amcor Road, Lyttleton Manor, Gauteng, South Africa
Emmanuelle Pourcher	IRB Services, 372 Hollandview Trail, Suite 300, Aurora, Ontario, Canada
Robert Prowse	Royal Adelaide Hospital Research Ethics Committee, Level 3, Hanson Institute, Royal Adelaide Hospital, North Terrace, Adelaide, South Australia
Pilar Quilez	Terrassa, Barcelona, Spain
Pilar Quiroga	Concepcion, Chile
Marina Raikhel	Copernicus IRB, USA
Ashok Raj	Copernicus IRB, USA
Surinder Randhawa	Copernicus IRB, USA
Seppo Rapo	Copernicus IRB, USA
Ramit Ravona-Springer	Sheba Medical Center EC, Tel Hashomer, Ramat Gan, Israel
Edgardo Gabriel Reich	Comité Independiente de ética para Ensayos en Farmacología Clínica, J.E. Uriburu 774-Piso 1, Ciudad Autónoma de Buenos Aire, Argentina

Investigator Name	IRB and/or IEC Name and Address
Anne Remes	Northern Savo Hospital District, Research Ethics Committee, PL 1777, Rakennus 4, Kuopio, Finland
Brigid Reynolds	Georgetown University Medical Center IRB, 3900 Reservoir Road, SW 104 Medical Dental Bldg, Washington, DC. USA
Philippe Robert	CPP SOOM 1, Place du Dr. Baylac, Bâtiment MNH – TSA 40031, Toulouse, Haute Garonne, France
Gustav Rohde	Comité Ético Científico SS Viña del Mar, Alvarez 1532 Piso 2 oficina 3, Viña del Mar, Chile
Graciella Rojas	Comité Ético Científico SSMCentral, Santa Rosa 1234, Santiago, Chile
Paul Rosenberg	Johns Hopkins IRB, 1620 McElderry Street, Reed Hall 130-B, Baltimore, MD, USA
Paolo Maria Rossini	Comitato di Bioetica Ospedale "S.Giovanni Calibita,Fatebenefratelli", Isola Tiberina, 39, Roma, Italy
Francois Rousseau	Comite d'ethique de la recherche du Centre Hospitalier Robert Giffard, 2601 chemin de la Canardière, Beauport, Quebec, Canada
Dan Rujescu	Uni Göttingen, Robert-Koch-Strasse 40, Goettingen, Germany
Marwan Sabbagh	Sun Health IRB, 10515 West Santa Fe Dr, Sun City, AZ, USA
Shoji Sakuragi	Tokushima University Hospital IRB, 2-50-1 Kuramoto-cho, Tokushima, Tokushima-Ken, Japan
Gustavo Saredo	Comité Independiente de ética para Ensayos en Farmacología Clínica, J.E. Uriburu 774-Piso 1, Ciudad Autónoma de Buenos Aire, Argentina
Ramanathan Sathianathan	EC Madras Medical College & Govt. Gen. Hospital, Chennai, Tamilnadu, India
Frederick Schaerf	Copernicus IRB, USA
Douglas Scharre	Western IRB, USA
Eugen Schlegel	Uni Göttingen, Robert-Koch-Strasse 40, Goettingen, Germany
Raymond Schwartz	NSCCH Human Research Ethics Committee (Harbour & Hawkesbury), Level 4, Vindin House Royal North Shore Hospital, St. Leonards, NSW

Investigator Name	IRB and/or IEC Name and Address
Daniel Seinhart	Comité Independiente de ética para Ensayos en Farmacología Clínica, J.E. Uriburu 774-Piso 1, Ciudad Autónoma de Buenos Aire, Argentina
Salumu Selemani	Pharma Ethics, 123 Amcor Road, Lyttleton Manor, Gauteng, South Africa
Arun Shah	Ethics Committee of B. Y. L. Nair Charitable Hospital, Pharmacology Dept 2nd floor college Building, Mumbai, India
Nellikunja Shankar	Mallikatta Ethical Committee, Ramkrishna Complex, Malikkatta, Kadri, Mangalore, India
Hideto Shinno	Kagawa University IRB for Clinical Study for Drugs and etc., 1750-1 Ikenobe,Miki-cho, Kita-gun, Kagawa-Ken, Japan
Alan Siegal	Copernicus IRB, USA
Richard Singer	Copernicus IRB, USA
Andrea Slachevsky	Comité Ético Científico SSMO, Av. Salvador 364, Providencia, Santiago, Chile
Joy Snider	Washington University IRB, St. Louis, MO. USA
Hilkka Soininen	Northern Savo Hospital District, Research Ethics Committee, PL 1777, Rakennus 4, Kuopio, Finland
Paul Solomon	Copernicus IRB, USA
Mary Stedman	Copernicus IRB, USA
Robert Stern	Boston University IRB, Boston, MA, USA
John Stoukides	Copernicus IRB, USA
Thatikonda Sudhakar	Institutional Ethics Committee, S. V. Medical College, Tirupati, Andhra Pradesh, India
Norio Taniguchi	Asakayama General Hospital IRB, 3-3-16 Imaike-cho Sakai-ku, Sakai, Osaka-Fu, Japan
Pierre Tariot	Western IRB, USA
Stephen Thein	Biomedical Research Institute of America, 2525 Camino del Rio South, Suite 300, San Diego, CA. USA

Investigator Name	IRB and/or IEC Name and Address
Alan Thomas	Cambridger 4 Research Ethics Committee, Victoria House, Capital Park, FULBOURN, Cambridge, UK
Therese Treves	Rabin Medical Center EC, 39 Jabotinsky St., Petah Tikva, Israel
Christopher Van Dyck	Yale University IRB, 47 College Street, Suite 204, New Haven, CT. USA
Marc Van Orshoven	Commissie Medische Ethiek van de UZ/KULeuven, Herestraat 49, Leuven, Belgium
Rik Vandenberghe	Commissie Medische Ethiek van de UZ/KULeuven, Herestraat 49, Leuven, Belgium
Chris Verster	Pharma Ethics, 123 Amcor Road, Lyttleton Manor, Gauteng, South Africa
DJ Volschenk	George, West Cape, South Africa
Anders Wallin	Regionala Etikprövningsnämnden i Stockholm, FE 289, Stockholm, Sweden
Jeanette Wendt	Copernicus IRB, USA
David Wilkinson	Cambridger 4 Research Ethics Committee, Victoria House, Capital Park, FULBOURN, Cambridge, UK
Michael Woodward	Austin Health Human Research Ethics Committee, Research Ethics Unit, Level 8 HSB - Room 8322, Austin Hospital, Studley Road, Heidelberg, Victoria, Australia
Jaroslaw Wronka	Komisja Bioetyczna przy ORL Wielkopolskiej Izby Lekarskiej, Nowowiejskiego 51, Poznan, Poland
Hideki Yamamoto	Kondo Hospital IRB, 4-114 Showadori, Amagasaki-shi, Hyogo-Ken, Japan
Yuval Zabar	Lahey Clinic IRB, 41 Mall Road, Burlington, MA, USA
Edward Zamrini	University of Utah IRB, USA
Earl Zimmerman	Western IRB, USA

Patient Information and Consent Form

Effect of γ-Secretase Inhibition on the Progression of Alzheimer's Disease: LY450139 versus Placebo

1.1. Introduction

You are invited to take part voluntarily in a research study of an investigational study drug known as LY450139. The study protocol has been amended due to the recent stopping of dosing. Your participation in this amended study is expected to last up to 32 weeks from the time of the dose stop. Up to 1500 patients will be participating in this study.

Eli Lilly and Company is sponsoring this study. Before agreeing to participate in this research study, it is important that you read and understand this form. It describes the purpose, procedures, benefits, risks and discomforts, and precautions of the study. It also describes the alternative procedures that are available to you and your right to withdraw from the study at any time. If you participate, you will receive a copy of this form to keep for your records.

We will ask you to come to all study visits with a study partner or caregiver. Your study partner or caregiver is someone who knows you well and will validate information we gather at your visits. It is important that you and your study partner/caregiver understand the description of the research study before you agree to participate.

In this consent form, "you" always refers to the patient. If you are a legally acceptable representative or study partner, please remember that "you" refers to the study patient.

1.2. Purpose of the Study

The primary purpose of your participation in this study is to help answer the following research question(s), and not to provide you treatment for your condition:

- The safety of LY450139 and any side effects that might be associated with it.
- To determine if the greater cognitive and functional worsening seen in LY450139-treated patients versus placebo-treated patients in the interim and futility analyses persists after LY450139 is discontinued.
- To monitor effects on concomitant medications following LY450139 cessation and to allow for concomitant medication adjustment, given the potential for LY450139 cessation to affect concomitant medication exposure.
- To monitor other safety measures following cessation of LY450139/placebo treatment through assessment of adverse events (AEs), routine physical/neurologic/skin examinations, and changes in vital signs, laboratory evaluations, and electrocardiograms (ECGs).

1.3. Qualifications to Participate

The doctor in charge of this study or a member of the study staff has discussed with you the requirements for participation in this study.

You cannot participate in this study if:

- you have a history of HIV;
- you have a history within the last 5 years of alcohol or drug abuse;
- you have a history within the last 5 years of a serious infection affecting the brain;
- you have multiple or severe drug allergies;
- you have serious or uncontrolled illnesses including those that affect your heart, kidneys, lungs, blood, liver, brain, nerves, ability to fight off infection, bowels stomach, mental health, abnormal blood/urine tests or other condition that the investigator feels could affect your participation in the trial;
- you cannot swallow a whole tablet;
- you are taking certain medications or may need to use certain medications during the study that are not allowed in this trial;
- you have uncontrolled high blood pressure.

If female, in order to participate in this study you must be post-menopausal, as evidenced by a lack of menstruation for at least 12 consecutive months or have had both ovaries removed.

It is important that you are completely truthful with the study doctor and staff about your past medical history as well as any symptoms experienced during the study. It may be harmful to you or to other people who may take the drug if you are not truthful with the study doctor and staff. You should not participate in this study if you do not meet all qualifications.

1.4. Study Procedures

If you decide to take part in this study, you will need to visit the hospital or study doctor's office at most 4 times over the 32 weeks from the dose stop. The first visit will be approximately 4 weeks after the dose stop. The second visit will be 8 weeks after the dose stop. The third visit will be 16 weeks after the dose stop. The fourth & final visit will be 32 weeks after the dose stop. Your study doctor or nurse will make sure you know when you need to attend.

You will have a number of different examinations and tests during the study at different visits. You will be asked questions about what other medications you are taking and how well you are feeling. Safety measures such as blood pressure, pulse, and electrocardiograms will also be taken. At all visits, you will have blood and urine samples taken. You will have a total-body skin examination at all visits. You will also be asked to answer questionnaires at study visits. The study visits will last up to 6 hours, depending on the evaluations made.

A blood sample will be taken at 4 visits during the study. The doctor or nurse will take your blood (approximately 2 tablespoons) for testing by placing a needle inside a vein in your arm. This will cause a small hole or "puncture". The blood samples will be collected to perform the

following tests: chemistry (to make sure the contents in your blood are within normal range), hematology (to make sure the levels of the different kinds of blood cells in your blood are normal) and drug monitoring tests (if you are taking certain medications certain blood tests will be performed to look for any changes in how it may be working). A urine sample will be taken at 4 visits during the study. You will be asked to urinate or “pee” into a small cup. A urinalysis (test of the urine) will be done to make sure the contents of your urine are within normal range.

Blood and urine samples collected for specified laboratory tests will be destroyed within 60 days of confirmation of the test results, unless laws, regulations, or international laboratory certification standards require a longer retention period. This confirmation will either occur immediately after initial testing, or may require that samples be held to be retested at a defined later point in time.

Certain samples (plasma) are being collected so that the sponsor can measure the amount of Aβ (a protein linked to Alzheimer’s disease) that is in your body and/or protein binding. The amount of blood not used for these tests will be stored. The sponsor will keep these samples with the patient identifier for a maximum of 2 years following last patient visit for the study. At the end of the 2 years, any remaining samples will be discarded.

Some samples of plasma will be stored for possible future research related to Alzheimer’s disease. Storage of these identifiable samples is a mandatory part of this study. These stored samples will keep the patient identification number and, therefore, will not be stored indefinitely. These samples will be stored for a maximum of 8 years after last patient visit for the study; any sample remaining at that time will be destroyed.

Please read the pages(s) called Study Procedures (Attachment 1). This will give you detailed information about how often you have to come to see the doctor/nurse, how long each visit may take how much blood will be taken and when tests and procedures will be performed on each study visit.

1.5. Risks

There may be risks to you if you participate in this study. LY450139 is new and has been taken only by a limited number of people. As of 07 September 2010, 367 people (297 healthy subjects and 70 people with Alzheimer’s disease [AD]) have taken semagacestat (LY450139) in completed studies.

1.5.1.1. Risks and Discomforts Associated with Semagacestat

An evaluation of ongoing study data showed that while patients taking placebo (sugar pill) showed worsening in memory and ability to perform routine activities at about the rate expected for Alzheimer’s patients, memory and function worsened more in patients taking semagacestat. Therefore, Lilly has decided to permanently stop study drug dosing in all semagacestat studies. For safety reasons, Lilly will continue to do follow-up testing for 7 months after stopping semagacestat. Because the studies are ongoing, the data are still blinded to you, your investigator, and the Lilly study team.

The following risks and discomforts have been noted in people taking semagacestat in other studies but most of these risks should diminish once you stop semagacestat.

A total of 70 people with AD have taken semagacestat in 2 completed clinical studies, and 297 healthy volunteers have taken semagacestat in 14 completed studies. The most common side effects are listed below.

**Of the 70 people with AD:**	
10 to 14 people reported:	• Diarrhea (unusually loose or frequent stools)<br>• Weakness.
5 to 9 people reported:	• Nausea (feeling sick to the stomach)<br>• Headache<br>• Vomiting<br>• Cough<br>• Rash<br>• Dizziness<br>• Sleepiness<br>• Confusion<br>• Back pain<br>• An infection of breathing system such as a cold or bronchitis

Of 108 healthy volunteers who took more than one dose of semagacestat:	
28 people reported:	• Headache
10 to 16 people reported:	• Rash<br>• Itchiness<br>• Diarrhea
5 to 9 people reported:	• Nausea<br>• Stomach pain<br>• Constipation (unusually hard or infrequent stools)<br>• Stomach or intestinal gas<br>• Sore throat<br>• Visual disturbances (such as blurred vision or seeing brief flashes of light)<br>• Dizziness<br>• Tiredness<br>• Back pain<br>• Indigestion<br>• Muscle pain<br>• Vomiting<br>• Throat irritation

Of 169 healthy volunteers who took just 1 dose of semagacestat:	
32 people reported	• Headache
11 people reported:	• Back pain
5 to 9 people reported:	• Pain in extremity<br>• Diarrhea<br>• Nausea<br>• Dizziness<br>• Neck pain<br>• Muscle spasms

One person with Alzheimer's disease was hospitalized for blockage of the intestine.

Five people (3 with Alzheimer's disease, 2 without) reported signs of blood in the stool or black stool, which may suggest injury to the stomach or intestines.

In animals and in a few people, hair color lightened. This unusual event happened after taking the drug for several weeks. This went away after people and animals stopped taking the drug.

Temporary changes in electrocardiograms (heart-trace readings) were seen in people taking daily doses of semagacestat. Changes in heart-trace readings may indicate a change in heart function that could be serious or life-threatening.

Skin cancer has been seen in some people with Alzheimer's disease taking daily doses of semagacestat or placebo (sugar pill) in 2 ongoing studies. Since these studies are still ongoing,

details about the rate of skin cancer with semagacestat are not yet available. Your skin will be checked for changes while you are participating in studies with semagacestat.

Semagacestat may change the effectiveness of some medicines by raising or lowering the amount in your blood. If you take one of these medicines, levels of these medicines in your blood and/or their effectiveness will be monitored in studies with semagacestat to be sure you are getting the right amount of medicine.

Some people taking medicines that lower a brain protein thought to cause Alzheimer's disease have experienced an increase in the water content of the brain tissue. Semagacestat works by lowering this brain protein, but no cases of an increase in the water content of brain tissue have been reported in patients taking semagacestat. Symptoms that can happen if someone develops this condition include headache, worsened confusion, change in level of consciousness, seizures, unsteadiness, and vomiting. If you develop a combination of these symptoms at the same time, you will be asked to get a scan of your brain. This would show whether the condition was present.

Bad effects in animals can indicate possible risks to humans. A higher rate of cancer of the ovaries and cancer of the uterus was seen in old animals that were given semagacestat every day from the time they were young compared with animals not given semagacestat. The effect on ovaries was likely related to the effect of semagacestat on young, active ovaries. The effect on the uterus might also be related to effects on ovaries and changes in hormones that might occur because of that. While the relevance to humans is not completely understood, human females taking semagacestat must be post-menopausal (that is, have had no menstrual periods for at least 12 months in a row or have had both ovaries removed), so their ovaries will no longer be active when they start taking semagacestat. Including only post-menopausal women is likely to reduce the risk. Ovarian or uterine cancer has not been reported with semagacestat in any of the human studies done so far.

Some animals given semagacestat had problems with the digestive system. For example, some had soft stools or soft stools with blood in them. Also, some animals had shrinking of some organs (including the spleen, prostate, adrenal glands, tear glands, uterus, and saliva-producing organs) and irritation of the liver. These drug effects disappeared when semagacestat was stopped. Based on certain effects seen in animals, infection may be more likely.

1.5.1.2. Effects on Male Fertility

A study of rats given semagacestat every day found that semagacestat may decrease male fertility. No change in the rat testicles was seen. The researchers think that the decreased fertility might be due to changes in semen. The effect on fertility in male rats went away after dosing was stopped. The effect of semagacestat on human male fertility is unknown.

1.5.1.3. Risks in Premenopausal Women

Semagacestat may affect reproduction. Studies in female dogs sometimes showed problems with ovary functioning. This could cause early menopause or infertility. Studies in pregnant rats and rabbits sometimes resulted in loss of the unborn babies, low- weight babies, or babies that did not

grow right. Women in the study must be post-menopausal, so these experiences will not affect women in the study.

Women who give semagacestat to patients are protected from the drug by a coating over the drug tablet. To keep safe, do not break the tablets into pieces or crush them. Broken tablets should be thrown away or returned to the study site in a sealed bag. If you touch a broken tablet, wash your hands right away to remove any drug material on your skin. Use a wet disposable towel to clean up dry, crushed tablets. If possible, wear disposable gloves when cleaning up spilled material.

1.6. Study Procedure Related Risks

Electrocardiograms (ECGs) (electrical tracings of the heartbeat or heart rhythm) will be done during this study in which you will have pads placed on different parts of your body. There is no pain or discomfort during an ECG; however, removing the pads may cause some irritation to your skin.

For most people, needle punctures for blood draws do not cause any serious problems. However, they may cause bleeding, bruising, discomfort, infections, dizziness, and/or pain at the needle site.

In addition to the risks already described, the study procedures may have other unknown risks.

At any time during this study, you may experience or worsening of your Alzheimer's disease. If you are using other approved treatments for Alzheimer's disease before entering the trial, you may be able to continue using them during the study. The study doctor will discuss this with you. There may be unknown risks of possible harmful interaction with other medication you may be taking.

1.7. Reporting Health Experiences

If you have any injury, bad effect, or any other unusual health experience during this study, make sure that you immediately tell the nurses or Dr. [study physician's name], at [phone #]. You can call at any time, day or night, to report such health experiences.

Other Treatments

You do not have to take part in this study to be treated for your illness or condition. Other treatments and therapies for your condition are available. Those might include cholinesterase inhibitors such as donepezil (Aricept®) and an *N*-methyl-D-aspartate receptor antagonist, memantine (Namenda®). The study doctor can discuss these treatments and therapies with you.

Participation in the Study

If any important new information is found during this study that may affect your wanting to continue to be part of this study, you will be told about it right away.

Your taking part in this study is entirely voluntary. You may refuse to take part in the study or you may stop your participation in the study at any time, without a penalty or loss of benefits to which you are otherwise entitled.

Your participation may also be stopped by the study doctor or sponsor without your consent. If you stop being part of this study, the study doctor or one of the staff members will talk to you about any medical issues regarding the stopping of your participation.

1.8. Treatment and Compensation for Injury

If you follow the directions of the study doctor and staff and you are physically injured due to any substance or procedure properly given under the plan for this study, the sponsor will pay the medical expenses for the treatment of that injury that are not covered by your medical insurance, by a government program, or by any other third party.

1.9. Possible Benefits

Study procedures will be provided at no cost to you.

You and your study partner/caregiver will be paid [insert amount per study visit] to reimburse you for transportation, parking, meal, or others expenses related to your participation in this study. If you withdraw from the study early, you will be paid for these expenses for the portion of the study that you did complete.

Information obtained from this study will benefit the sponsor of the study, Eli Lilly and Company, and may benefit subjects/patients in the future.

You may receive information about your health from any physical examinations and laboratory tests to be done in this study.

1.10. Investigator Payment

The sponsor is paying the study doctor and/or [name of Institution or site] for their work in this study.

Questions

If you have any questions about this study or your rights, please contact Dr. [study physician's name] at [address and phone #].

If you have any questions about your rights as a participant in a research study, please contact [ERB contact or other neutral or disinterested party] at [address and phone number].

Confidentiality

The study doctor and staff will handle your personal health information in a confidential manner. Your health information will be used and disclosed in accordance with the following U.S. Data Privacy Statement.

U.S. Data Privacy Statement

A federal government rule has been issued to protect the privacy rights of subjects/patients. This rule was issued under a law called the Health Insurance Portability and Accountability Act of 1996 (HIPAA). This rule is designed to protect the confidentiality of your personal health information. Your personal health information is information about you that could be used to find out who you are. For this research study, this includes information in your existing medical records needed for this study and new information created or collected during the study.

This Data Privacy Statement explains how your personal health information will be used and whom it will be given to ("disclosed") for this research study. It also describes your privacy rights, including your right to see your personal health information.

By signing the consent document for this study, you will give permission ("authorization") for the uses and disclosures of your personal health information that are described in this Data Privacy Statement. If you do not want to allow these uses, you should not participate in this study.

If you agree to participate in the research study, your personal health information will be used and disclosed in the following ways:

The study doctor and staff will use your medical records and information created or collected during the study to conduct the study.

The study doctor and staff will send your study-related health information ("study data") to the sponsor of the study and its representatives ("sponsor"). Because the sponsor conducts business related to clinical research in many countries around the world, this may involve sending your study data outside of the United States. Other countries may have privacy laws that do not provide the same protections as the laws in this country. However, the sponsor will respect the terms of this Data Privacy Statement in all countries.

The study data sent by the study doctor to the sponsor does not include your name, address, social security number, or other information that *directly* identifies you. Instead, the study doctor assigns a code number to the study data and may use your initials. Some study data sent to the sponsor may contain information that could be used (perhaps in combination with other information) to identify you (e.g., date of birth). If you have questions about the specific health information that will be sent to the sponsor, you should ask the study doctor.

The sponsor will use the study data for research purposes to support the scientific objectives of the study described in the consent document, to assess the safety or efficacy of any drug or treatment included in the study, to better understand the disease(s) included in the study, or to improve the design of future studies.

Your study data, either alone or combined with data from other studies, may be shared with regulatory authorities in the United States and other countries, doctors at other institutions participating in the study, and the ethical review board overseeing this study.

Study data that does not directly identify you may be published in medical journals or shared with others as part of scientific discussions.

Your original medical records, which may contain information that directly identifies you, may be reviewed by the sponsor, the ethical review board overseeing this study, and regulatory authorities in the United States and other countries. The purpose of these reviews is to assure the quality of the study conduct and the study data, or for other uses authorized by law.

The sponsor works with business partners in drug development. The sponsor may share your study data with these business partners, but only if the business partners need the information as a part of this work with the sponsor, and only if the business partners signs a contract that requires it to protect your study data in the same way as the sponsor.

The sponsor will not disclose personal health information to insurance companies unless required to do so by law, or unless you provide separate written consent to do so.

Your medical records and study data may be held and processed on computers.

Your personal health information may no longer be protected under the HIPAA privacy rule once it is disclosed by your study doctor to these other parties.

You have the right to see and copy your personal health information related to the research study for as long as this information is held by the study doctor or research institution. However, to ensure the scientific integrity of the study, you will not be able to review some of the study information until after the study has been completed.

You may cancel your authorization at any time by providing written notice to the study doctor. If you cancel your authorization, the study doctor and staff will no longer use or disclose your personal health information in connection with this study, unless the study doctor or staff needs to use or disclose some of your personal health information to preserve the scientific integrity of the study. The sponsor will still use study data that was collected before you cancelled your authorization. If you cancel your authorization, you will no longer be able to participate in the study. However, if you decide to cancel your authorization and withdraw from the study, you will not be penalized or lose any benefits to which you are otherwise entitled.

Your authorization for the uses and disclosures described in this Data Privacy Statement does not have an expiration date.

Patient Information and Consent Form
Attachment 1

Signature Pages

To be entered into the study, you or a legal representative in addition to your caregiver must sign and date the signature pages which follow. The following are required

- Name and signature of participant OR legally authorized representative.
- Name and signature of witness to participant signature. (If applicable / required by IRB/EC. This is not a Lilly requirement.)
- Name and signature of caregiver
- Name and signature of person conducting consent discussion.
- Date of signature for each individual (personally completed)

By signing below, you are confirming the following:

- You have read all of the information in this Patient Information and Consent Form, and you have had time to think about it.
- All of your questions have been answered to your satisfaction.
- You voluntarily agree to be part of this research study, to follow the study procedures, and to provide necessary information to the study doctor, nurses, or other staff members, as requested.
- You may freely choose to stop being a part of this study at any time.
- You allow the study doctor and the sponsor to use and disclose your personal health information as described in this document.
- You have received a copy of this signed Patient Information and Consent Form to keep for yourself.

FOR SUBJECT/PATIENT TO COMPLETE

Signature of Patient	Date
Patient Name (print)	Patient Number

FOR LEGAL REPRESENTATIVE TO COMPLETE

Signature of Legal Representative	Date
Legal Representative Name (print)	
If signed by legal representative, state description of relationship to subject/patient or other basis for legal authority	

FOR INDIVIDUAL CONDUCTING INFORMED CONSENT

Signature of individual conducting informed consent discussion	Date
Name of individual conducting informed consent discussion (print)	

SIGNATURE OF IMPARTIAL WITNESS (IF APPLICABLE)_

Signature of Impartial Witness	Date
Impartial Witness Name (print)	Patient Number

(If page not applicable: line through, date & initial)

If the patient does not have the capacity to give informed consent to participate in this research study, the legal representative must read and sign the following affidavit:

By signing below, I ___________________________ certify that I have read or had this

(Print legal representative's name)

document read to me. I will be given a signed copy. I have been given the chance to ask questions and to discuss the patient's participation with the investigator. As legal representative for this patient, I hereby give consent for this patient to participate in this study. I conclude that the patient wishes to participate in this research and has indicated his/her assent to do so. (I do/do not have durable power of attorney for health care for the patient. I am/am not the patient's legal guardian/representative.)

____

Name of Legal Representative (Print)

____ ______________________________

Signature of Legal Representative Date

____ ______________________________

Signature of Subject Date

____ ______________________________

Signature of Investigator Date

____ ______________________________

Signature of Witness Date

Caregiver Information

As the patient's caregiver, I have important responsibilities that need to be followed in order for the study to be conducted in the safest and best manner possible. These responsibilities are as follows:

1. I must accompany the patient to all clinic visits and must have direct contact with the patient for a minimum of three days per week (for at least 4 hours per day during waking hours).
2. I will receive a schedule for clinic appointments from the study doctor. I must try to help the patient keep all scheduled appointments.
3. I am a very valuable source of information about the patient. The study doctor will interview me during the clinic visits. My cooperation in answering these questions is necessary.

Caregiver Consent

I, ______________________, have read and understand all of the preceding information that describes the patient's participation in the study, my responsibilities as the patient's informant, and possible risks from exposure to broken tablets. I voluntarily consent to participate in the study. (I do/do not have durable power of attorney for health care for the patient. I am/am not the patient's legal guardian.)

FOR CAREGIVER TO COMPLETE

Signature of Caregiver	Date
Caregiver Name (print)	

FOR INDIVIDUAL CONDUCTING CAREGIVER INFORMED CONSENT TO COMPLETE

Signature of individual conducting caregiver informed consent discussion	Date
Name of individual conducting caregiver informed consent discussion (print)	

Patient Information and Consent Form
Attachment 2

Study Procedures

H6L-MC-LFAN(e)

**Endpoint Visit**	The Endpoint Visit is the completion of the protocol as originally designed.
**Approximate Visit Length**	Up to 6 hours
**Procedures To Be Done**	-Answer 7 questionnaires to evaluate memory and mood<br>-Neurological examination<br>-Physical examination, including body weight and vital signs (blood pressure, pulse, and temperature)<br>-You will have a total-body skin examination.<br>-Electrocardiogram (performed 3 times)<br>-Urine sample collected<br>-Blood sample (2 to 5 tablespoons) taken
**Study Visit 801**	This visit will occur for patients who choose to discontinue the study at or before the Endpoint Visit.
**Approximate Visit Length**	Up to 1 hour
**Procedures To Be Done**	-Electrocardiogram<br>-You will have a total-body skin examination.<br>-Blood sample (2 to 5 tablespoons) taken<br>-Answer questions about your general health
**Study Visits: 802, 803, and 804**	These visits will evaluate your general health and well being.
**Approximate Visit Length**	Up to 6 hours
**Procedures To Be Done**	-Answer 2 questionnaires<br>-Neurological examination<br>-Physical examination, including body weight and vital signs (blood pressure, pulse, and temperature)<br>-Answer questions about your general health<br>-Blood sample (2 to 5 tablespoons) taken<br>-Electrocardiogram<br>-Urine sample collected<br>-You will have a total-body skin examination.
**6-Month Post-Study Virtual Follow-up**	This visit will be a telephone or certified mail follow-up only if you discontinue before or at the Endpoint Visit.
